# Research trends and hotspots of radioiodine-refractory thyroid cancer treatment in the twenty-first century: a bibliometric analysis

**DOI:** 10.1007/s12149-024-01998-2

**Published:** 2024-11-05

**Authors:** Yuhang Xue, Yuzhe Zhang, Xintao Ding, Xinyu Wu, Bo Li, Ye Zhang, Yongju Gao

**Affiliations:** 1 Department of Nuclear Medicine, People’s Hospital of Zhengzhou University, Henan Provincial People’s Hospital, Zhengzhou, 450003 China; 2 The First Laboratory of Cancer Institute, The First Hospital of China Medical University, Shenyang, 110001 China; 3 Department of Biomedical Informatics, Columbia University Graduate School of Arts and Sciences, New York, NY USA; 4 Business and Strategy Analytics, Progyny, Inc., New York, NY USA

**Keywords:** Thyroid cancer, Radioiodine-refractory, Treatment, Bibliometric analysis, VOSviewer, Citespace

## Abstract

The treatment of radioiodine-refractory differentiated thyroid cancer (RAIR-DTC) has made significant advancements in the twenty-first century. This study aimed to assess the current state of research and identify potential new directions by conducting a bibliometric analysis of scientific publications on RAIR-DTC treatment. Publications relevant to RAIR-DTC, published from January 1, 2000, to December 31, 2023, were retrieved from the Web of Science Core Collection. Bibliometric analyses of major keywords, authors, countries, institutions, publications, and journals were conducted using CiteSpace and VOSviewer. A total of 859 papers were included in the analysis. The results demonstrated a rising trend in the number of publications over time. The United States was identified as the leading contributor in terms of publication output, citations, and international collaborations. Gustave Roussy emerged as the top organization in publication productivity, while the journal Thyroid had the highest number of related publications. The research on RAIR treatment was categorized into three key hotspots: clinical trials of targeted therapies, novel therapeutic strategies, and debates surrounding the RAIR-DTC management. RAIR-DTC research is expanding from the clinical trial phase of tyrosine kinase inhibitor monotherapy to a more complex combination therapy strategy, in particular, the synergistic effect of immune checkpoint inhibitors and other therapeutic agents, requiring more high-quality prospective studies to validate the clinical benefits. Moreover, the timely identification of RAIR-DTC patients holds the potential to enable early disease intervention, constituting a pivotal novel research direction in the future.

## Introduction

Thyroid cancer is the most common endocrine malignancy, with its incidence steadily rising in recent decades [1, 2]. Differentiated thyroid cancer (DTC), including papillary thyroid cancer (PTC) and follicular thyroid cancer (FTC), typically has a favorable prognosis [3]. These tumor cells retain the ability to take up and organify iodine, as well as secrete thyroglobulin (Tg) in response to thyroid-stimulating hormone (TSH) stimulation [4]. Due to these retained functions, most DTCs are curable through surgery and radioiodine (RAI) therapy, with 10-year survival rates exceeding 90% [5, 6]. However, approximately 15% of DTC patients experience a reduction or loss of iodine uptake, accompanied by decreased sodium iodide symporter (NIS) expression in the plasma membrane, rendering them resistant to RAI treatment [7, 8]. In such cases, the 10-year survival rate drops below 20% [9].

Despite the availability of various therapeutic options—such as surgical resection, external beam radiation therapy (EBRT), iodine-125 (^125^I) implantation, and chemotherapy—treating radioiodine-refractory differentiated thyroid cancer (RAIR-DTC) remains a significant clinical challenge due to the limited efficacy of these treatments and the potential for severe adverse effects [10].

With advancements in understanding the molecular mechanisms of thyroid cancer, sorafenib and lenvatinib have been approved by the US Food and Drug Administration (FDA) and the European Medicines Agency (EMA) for the treatment of RAIR-DTC [11]. However, these therapies require continuous dosing and are associated with significant toxicity [12]. A promising therapeutic approach is the pharmacologically induced re-expression of NIS, which allows tumor cells to regain the ability to respond to RAI treatment [11]. Studies have shown that various agents, including retinoids (vitamin A-derived retinoic acids), the MEK inhibitor selumetinib, and BRAF inhibitors such as vemurafenib and dabrafenib, can induce redifferentiation in thyroid cancer cells [13–16]. Additionally, immunotherapy is emerging as a potential treatment for refractory thyroid cancers, particularly advanced follicular cell-derived cancers and medullary thyroid cancer (MTC), marking a new frontier in the treatment landscape [17–19].

In recent decades, numerous studies have advanced both the basic research and clinical translation of RAIR-DTC treatments. However, a comprehensive assessment of the related literature is lacking. Bibliometric analysis is a valuable scientific method that applies mathematical and statistical techniques to quantitatively evaluate large volumes of research, identifying key trends and hotspots within a field [20, 21]. This study aims to conduct a bibliometric analysis and visualization of research on RAIR treatment, providing a theoretical foundation for future investigations in this area.

## Materials and methods

### Data collection

**Fig. 1:**
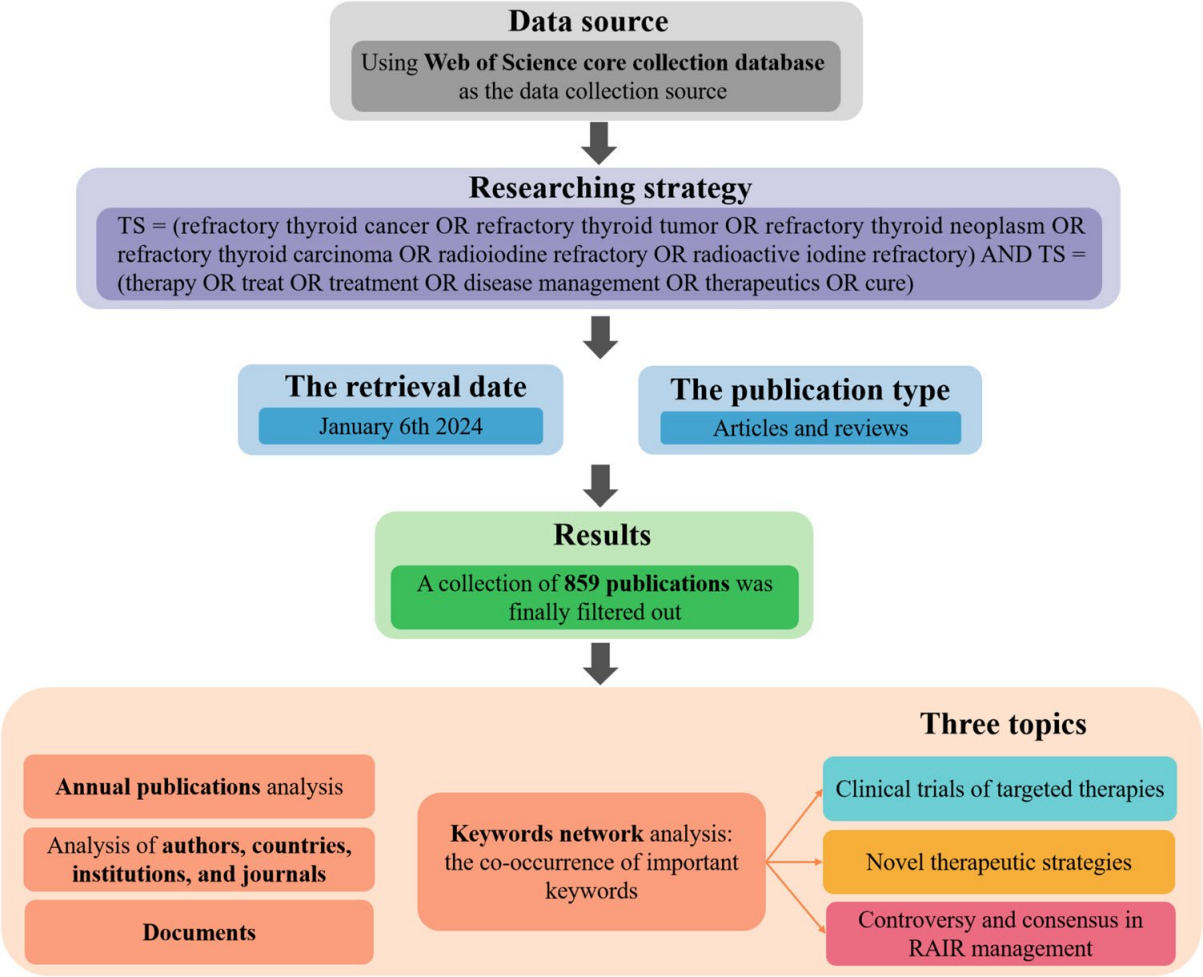
Flowchart illustrating the literature screening process

On January 6, 2024, we performed a comprehensive literature search on treatments for RAIR-DTC, covering studies from 2000 to 2023 using the Web of Science Core Collection (WoSCC). To reduce the risk of bias from database updates, all data were retrieved on a single day. The search strategy was designed as follows: TS = (refractory thyroid cancer OR refractory thyroid tumor OR refractory thyroid neoplasm OR refractory thyroid carcinoma OR radioiodine refractory OR radioactive iodine refractory) AND TS = (therapy OR treat OR treatment OR disease management OR therapeutics OR cure). The search was limited to publications classified as "Article" or "Review" and restricted to English-language documents. Two researchers independently checked and manually excluded publications that were not relevant to the topic. All search results were exported in TXT format as "full record and cited references". After duplicate entries were removed using CiteSpace, 859 documents were selected for bibliometric analysis (Fig. 1).

### Visualization tools

The present study utilized three visualization tools: VOSviewer (v.1.6.19), CiteSpace (v.6.2.R4), and Scimago Graphica (v.1.0.36). VOSviewer was used to construct and display scientific maps [22]. CiteSpace facilitated the analysis of citation networks, author collaborations, and topic evolution, helping to identify trends and research hotspots in the field [23]. Scimago Graphica was employed to map the global distribution of national publications, illustrating the international research landscape and collaboration dynamics [24].

## Results

### Annual publication analysis

**Fig. 2:**
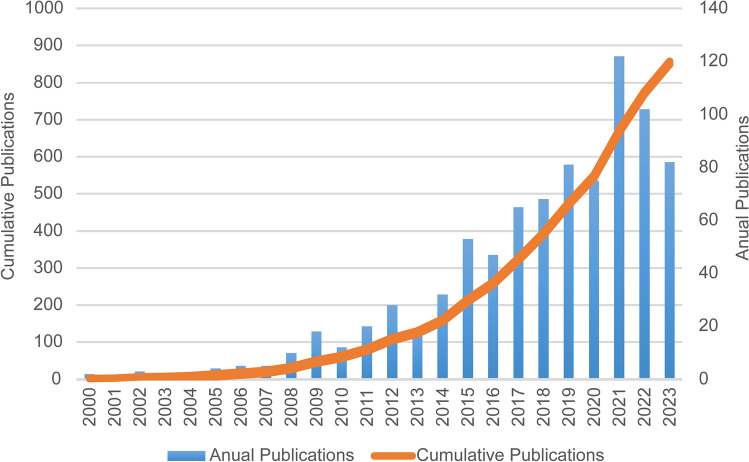
The number of articles published in the twenty-first century and the trend in the growth of the annual number of articles

Figure 2 presents the annual trend of publications related to RAIR therapy from 2000 to 2023. A total of 859 publications met the inclusion criteria, consisting of 637 articles and 222 reviews. Since 2000, there has been a sustained increase in the number of publications. Notably, in 2021, the number of articles reached 122, reflecting growing research interest and engagement in this field.

### Global publication and collaboration analysis

**Fig. 3:**
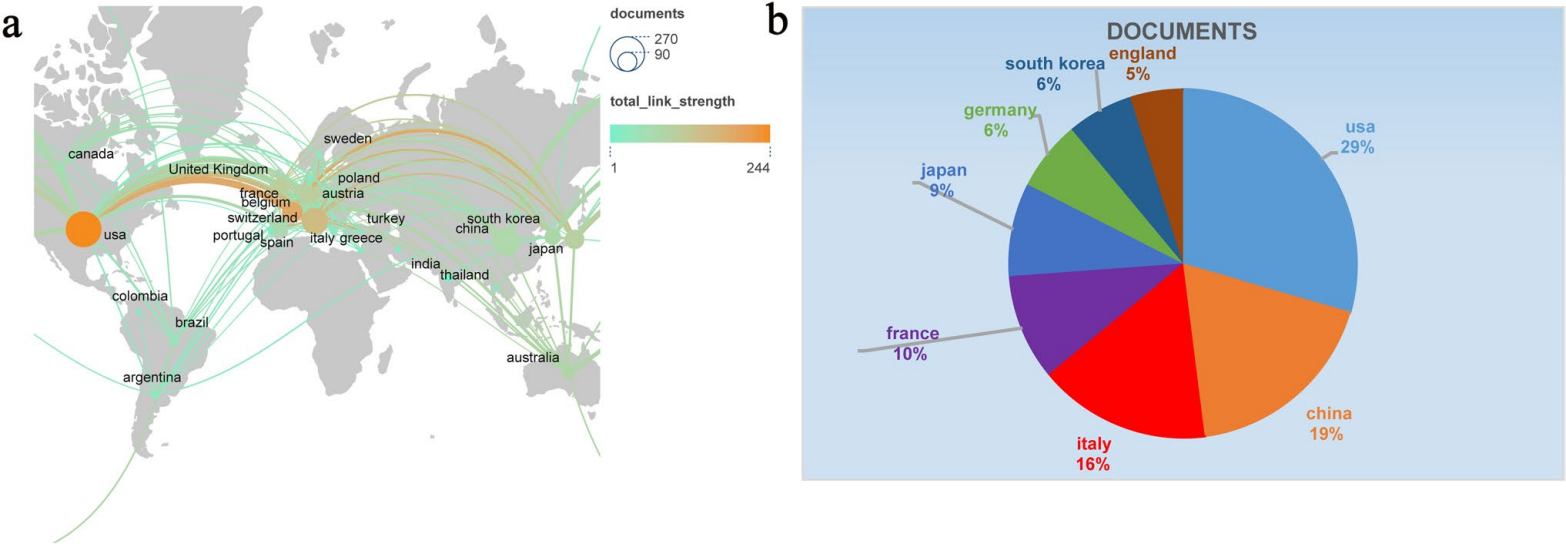
**a** World map depicting the number of publications by country. Circle size represents the volume of publications, while the thickness of the connecting lines indicates the strength of collaboration between countries. **b** The percentage of total publications contributed by the eight countries with the highest number of articles in the field

Figure 3a illustrates the global collaborative network of countries, highlighting significant research interest from European nations such as France, Germany, and Italy. The United States leads in publications, contributing 259 articles, which accounts for 30% of the total output. Figure 3b presents the distribution of articles among the top eight countries, with the United States, China, and Italy ranking as the top three contributors in terms of publication volume.

### Author and institutional analysis

**Fig. 4:**
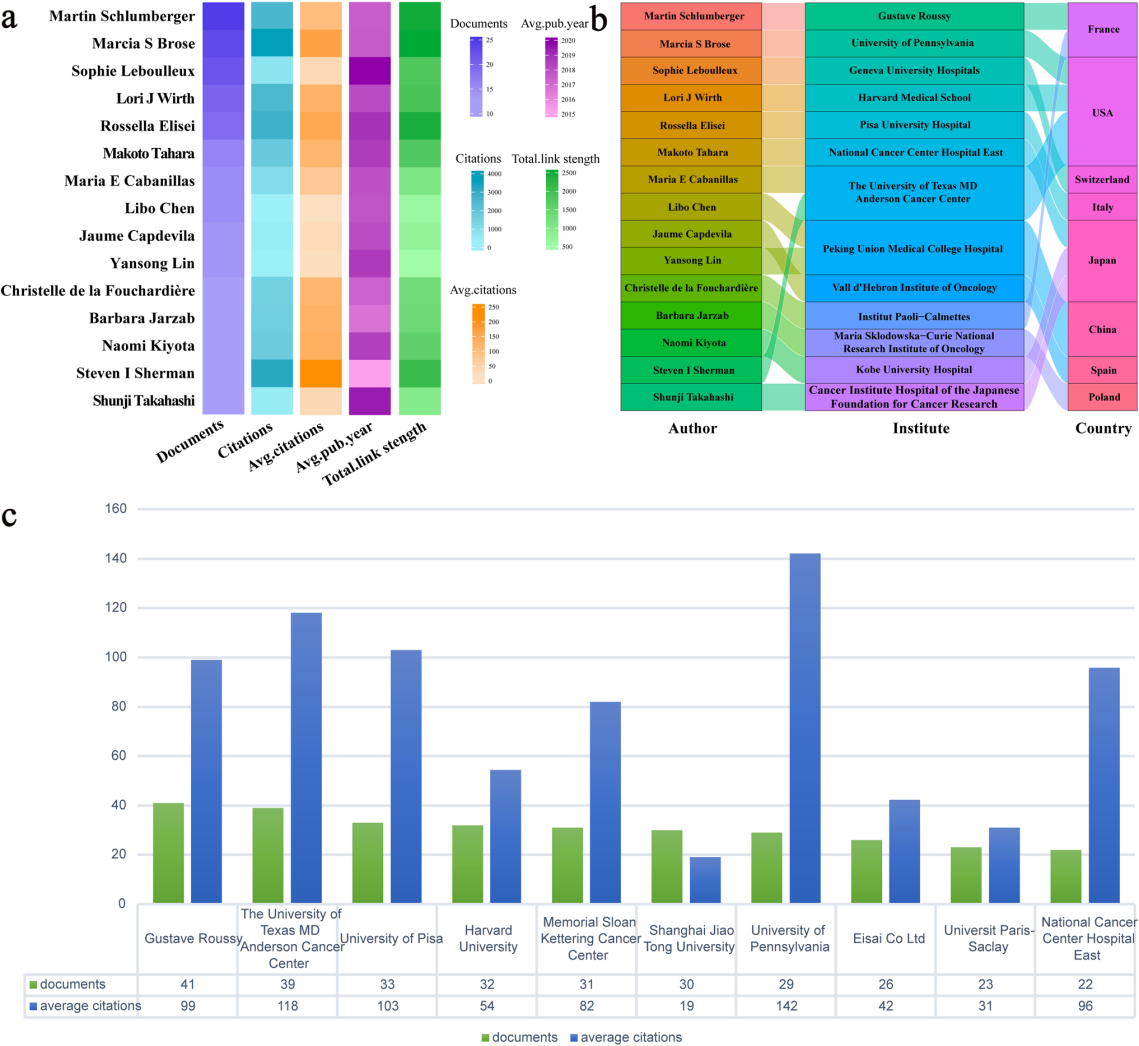
Analysis of the top 15 authors and 10 institutions by publication volume. **a** The top 15 authors' number of papers, total citations, average number of citations, intensity of collaboration, and average time to publication. **b** Institutional affiliation and country of the top 15 authors with the most publications. **c** Number of documents and average citations for the 10 most published institutions

The top 15 authors in terms of number of publications were analyzed. As shown in Fig. 4a, Martin Schlumberger leads with 26 publications. Marcia S. Brose holds the highest citation count, with 3806 citations, while Steven I. Sherman ranks second with 3214 citations and has the highest average citations per publication, approximately 247. Notably, Sophie Leboulleux's publications are the most recent on average, with a median publication year of 2020. Of the top 15 researchers, four are from the United States, and three are from Japan (Fig. 4b).

Among the top 10 institutions with the highest number of publications, Gustave Roussy ranks first in total publications, while the University of Pennsylvania has the highest average citations per publication (Fig. 4c).

### Core journal analysis

**Fig. 5:**
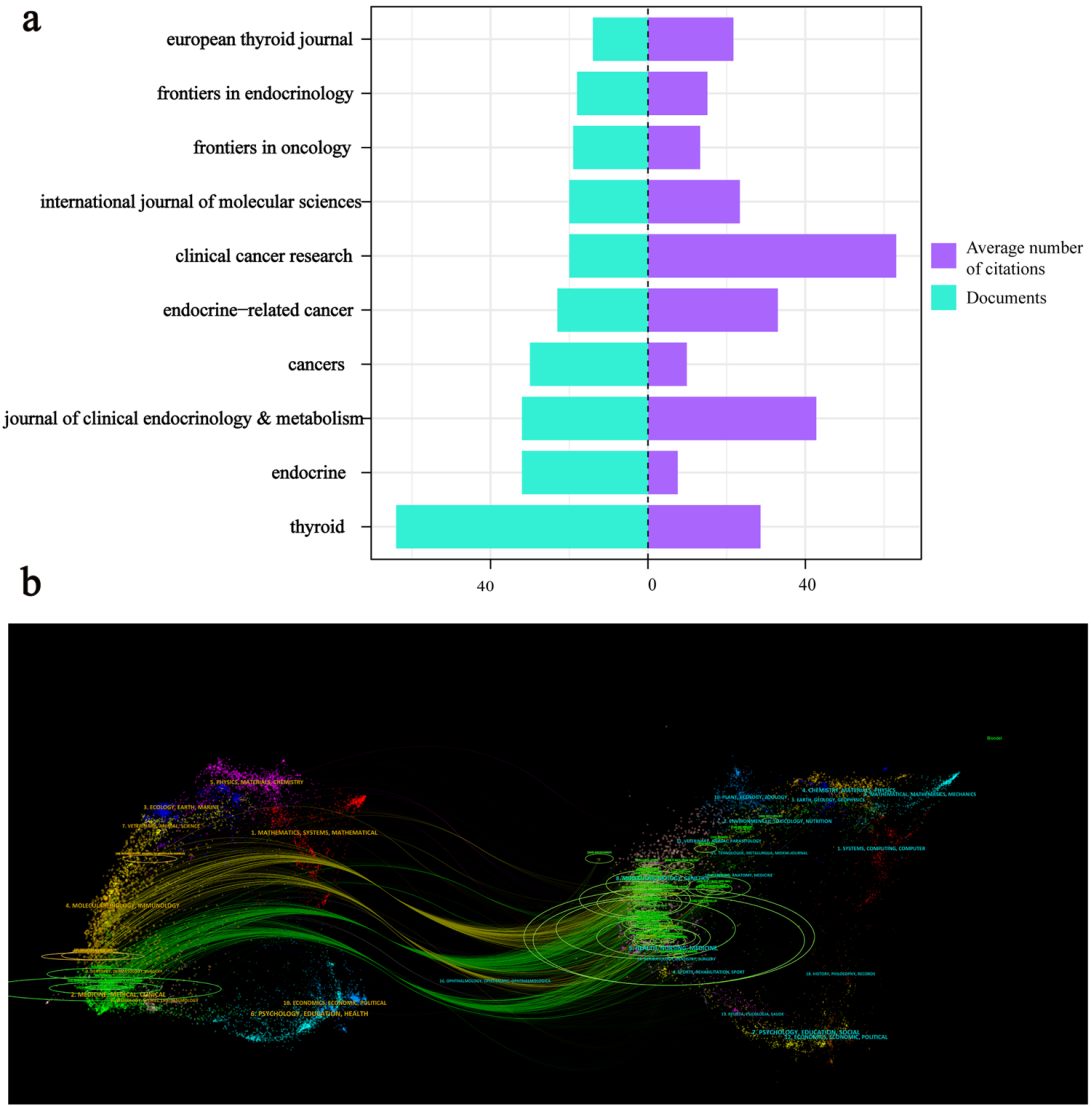
**a** Number of publications and average citations for the top 10 journals with the highest article counts. **b** Dual-overlay graph of journals in the field of RAIR-DTC treatment

Figure 5a highlights the top 10 journals publishing articles on RAIR treatment from 2000 to 2023, collectively contributing 272 articles, which represent 31.66% of the total publications. Among these, Thyroid ranked first with 64 papers, accounting for 7.10% of all publications. Articles in Clinical Cancer Research had an average of 63 citations, underscoring the journal's significant impact. The dual graph overlay (Fig. 5b) illustrates the citation pathways, with citing journals on the left and cited journals on the right. The published articles were mainly in the fields of medicine, medical, and clinical; while the cited publications predominantly came from health, nursing, and medicine.

### Highly cited articles analysis

**Fig. 6:**
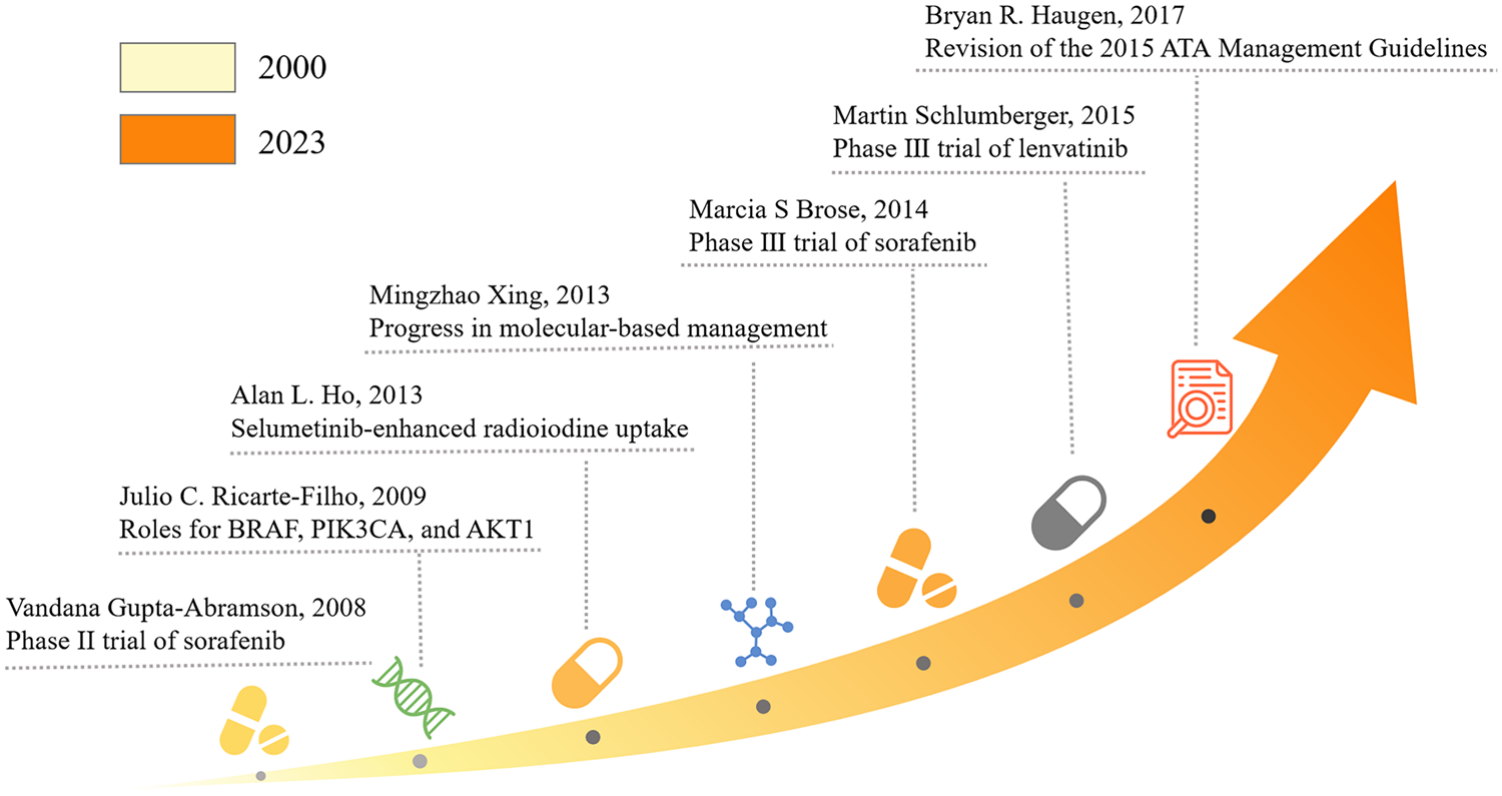
Chronological timeline of pivotal articles in RAIR-DTC treatment research

Table 1 lists the top 10 most cited articles. The most frequently cited paper, Lenvatinib versus placebo in radioiodine-refractory thyroid cancer, published in the New England Journal of Medicine in 2015, has been cited 1,220 times. Seven of the top 10 articles are clinical trial studies focused on targeted therapies for advanced, progressive, and RAIR-DTC. By systematically analyzing citation counts, we identified a series of pivotal studies and constructed a chronological timeline highlighting several milestone publications (Fig. 6) [14, 25–30].

**Table 1:** The top 10 co-citation references related to RAIR-DTC treatment

Rank	Year	First author	Title	Source	Citations	Type
1	2015	Martin Schlumberger	Lenvatinib versus placebo in radioiodine-refractory thyroid cancer	New England Journal of Medicine	1220	Clinical Trial
2	2014	Marcia S Brose	Sorafenib in radioactive iodine-refractory, locally advanced or metastatic differentiated thyroid cancer: a randomized, double-blind, phase 3 trial	Lancet	1024	Clinical Trial
3	2013	Alan L Ho	Selumetinib-enhanced radioiodine uptake in advanced thyroid cancer	New England Journal of Medicine	547	Clinical Trial
4	2008	Vandana Gupta-Abramson	Phase II trial of sorafenib in advanced thyroid cancer	Journal of Clinical Oncology	540	Clinical Trial
5	2013	Mingzhao Xing	Progress in molecular-based management of differentiated thyroid cancer	Lancet	405	Review
6	2009	Julio C Ricarte-Filho	Mutational profile of advanced primary and metastatic radioactive iodine-refractory thyroid cancers reveals distinct pathogenetic roles for BRAF, PIK3CA, and AKT1	Cancer Research	393	Research Article
7	2017	Bryan R Haugen	2015 American thyroid association management guidelines for adult patients with thyroid nodules and differentiated thyroid cancer: what is new and what has changed?	Cancer	348	Commentary
8	2010	Keith C Bible	Efficacy of pazopanib in progressive, radioiodine-refractory, metastatic differentiated thyroid cancers: results of a phase 2 consortium study	Lancet Oncology	326	Clinical Trial
9	2010	Laurie L Carr	Phase II study of daily sunitinib in FDG-PET-positive, iodine-refractory differentiated thyroid cancer and metastatic medullary carcinoma of the thyroid with functional imaging correlation	Clinical Cancer Research	316	Clinical Trial
10	2018	John D Hainsworth	Targeted therapy for advanced solid tumors on the basis of molecular profiles: results from MyPathway, an open-label, phase IIa multiple basket study	Journal of Clinical Oncology	296	Clinical Trial

### Keyword co-occurrence analysis

**Fig. 7:**
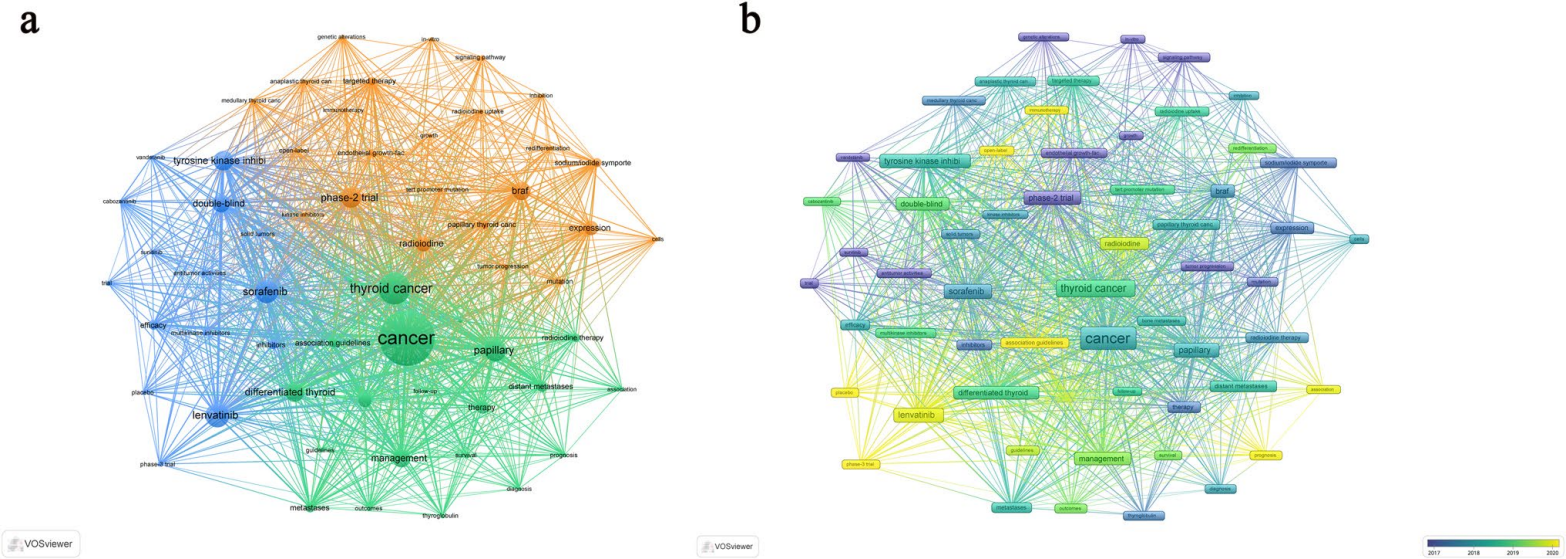
**a** Co-occurrence mapping of key terms, displaying 60 keywords with a minimum occurrence threshold of 5. **b** Distribution of keywords based on their average year of publication

Since the keywords condense the essence of an article, we can capture the main directions and hotspots in the field of RAIR-DTC comorbid with depression based on keyword co-occurrence analysis. Using VOSviewer, we set the "minimum number of occurrences per keyword" to 5 and the "total number of selected keywords" to 60. As shown in Fig. 7a, the keywords were grouped into three clusters: the blue cluster (clinical trials of targeted therapies), the yellow cluster (novel therapeutic strategies for RAIR-DTC), and the green cluster (debates surrounding the RAIR-DTC management). In Fig. 7b, the keywords are color-coded based on the average publication year (AAY), with yellow indicating the most recent publications. The latest keywords include "phase-3 trial," "placebo," "immunotherapy," "association guidelines," and "prognosis."

## Discussion

### Global research status and trends

Annual and cumulative publication trends in RAIR-DTC therapy show that over half of the relevant studies were published between 2019 and 2022, reflecting growing research interest and a continuous upward trajectory. National publication data reveal that the United States leads with 259 articles. The international collaboration map highlights strong cooperation between the United States and European countries. Notably, four of the top 15 most prolific authors are from the United States, underscoring the country's leadership in RAIR-DTC treatment research and its active engagement in global collaboration. Among institutions, Gustave Roussy had the highest publication output, while articles from The University of Texas MD Anderson Cancer Center demonstrated greater academic impact. Co-citation analysis identified Journal of Clinical Endocrinology & Metabolism and Thyroid as key journals in RAIR-DTC therapy research, and researchers are advised to closely monitor recent publications in these journals.

### Research hotspots on RAIR-DTC

Keyword cluster analysis provides a comprehensive understanding of the primary research directions and potential emerging trends, offering valuable insights into future areas of interest. From the co-occurrence mapping of keywords in the RAIR-DTC treatment field, the identified research hotspots were categorized into three distinct clusters, each representing a different research focus.

### Blue cluster: clinical trials of targeted therapies

The blue cluster highlights keywords such as "tyrosine kinase inhibitors," "sorafenib," and "lenvatinib," focusing on the development of targeted therapies, their safety profiles, and potential combination treatments.

Tyrosine kinase inhibitors (TKIs) exert antitumor effects by selectively inhibiting tyrosine kinases, which are involved in key signaling pathways that regulate cancer cell growth and survival [31]. Sorafenib and lenvatinib are the two most extensively studied TKIs in this field [25–27, 32–39]. In 2008, Gupta-Abramson et al. (R-4) conducted a phase II, single-arm trial to assess the efficacy of sorafenib in advanced thyroid cancer [27]. The trial included 30 patients, all treated for at least 16 weeks, and reported an overall clinical benefit rate (partial response + stable disease) of 77%, with a median progression-free survival (PFS) of 79 weeks. Based on the clinical efficacy of this study, Brose et al. (R-2) initiated a phase III trial, known as the DECISION trial (NCT01761266), to evaluate the efficacy of sorafenib in patients with locally advanced or metastatic RAIR-DTC [26]. The trial demonstrated a significant improvement in PFS for patients treated with sorafenib compared to placebo, leading to FDA approval of sorafenib for RAIR-DTC in 2013. Lenvatinib has also shown considerable clinical efficacy in RAIR-DTC treatment. Schlumberger et al. conducted the SELECT study, a randomized, double-blind, multicenter phase III trial involving 392 patients [25]. This study demonstrated that lenvatinib significantly prolonged PFS in patients with RAIR-DTC, with a median PFS of 18.3 months compared to 3.6 months in the placebo group. The objective response rate (ORR) in the lenvatinib group reached 64.8%. Due to its impact, this study has become the most cited paper in RAIR-DTC therapy research.

While TKIs such as lenvatinib and sorafenib offer significant clinical benefits, their use presents two major challenges: adverse effects and acquired resistance [11, 40–43]. A meta-analysis by Yu et al. found that patients treated with sorafenib experienced a higher incidence of hand-foot syndrome, hypocalcemia, rash, and elevated liver enzymes (ALT and AST) compared to those on lenvatinib [42]. In a broader analysis of drug therapies, Ji et al. reported that lenvatinib had the highest rate of severe adverse events (Grade ≥ 3), with hypertension (47.5%) being the most common, followed by proteinuria (13.7%), diarrhea (7.7%), and fatigue (6.6%) [40]. Another critical issue with TKIs is the development of acquired resistance, which limits their long-term efficacy. The mechanisms of resistance are diverse, with RET-dependent target modifications and bypass signaling being the most common [41]. Additionally, the tumor microenvironment and immune cell infiltration play pivotal roles in the development of resistance.

Given these challenges, recent research has increasingly focused on combination therapies to improve efficacy and mitigate resistance. For instance, the combination of sorafenib with everolimus (NCT01141309) is currently being investigated in progressive RAIR-DTC to assess potential synergistic effects [44]. Early stage trials have shown promising results, with the combination therapy group exhibiting better response rates and lower toxicity compared to sorafenib monotherapy. Additionally, the NCT01270321 trial delved into the efficacy of the combined administration of everolimus and pasireotide long-acting release in advanced thyroid cancer, revealing that this combinatorial approach yielded notably superior therapeutic outcomes in comparison to the utilization of the individual agents alone [45]. As these studies progress, combination therapies are expected to be further optimized, offering personalized, highly effective, and safer treatment options for patients with diverse genetic profiles.

### Yellow cluster: novel therapeutic strategies

The yellow cluster is defined by core keywords such as "BRAF," "sodium/iodide symporter," "redifferentiation," and "immunotherapy," representing research focused on dedifferentiation mechanisms and emerging therapeutic approaches.

RAIR-DTC has a high prevalence of BRAF mutations [46], which lead to activation of the MAPK pathway and suppression of NIS expression, resulting in reduced iodine uptake in thyroid cells [47–50]. Restoring the sensitivity of these tumors to radioiodine is a complex and critical subject. Chakravarty et al. demonstrated that MAPK pathway inhibitors can restore RAI uptake in a mouse model of DTC with the BRAFV600E mutation [46]. Building on this, Ho et al. (R-3) published a 2013 clinical trial using selumetinib, an MAPK kinase (MEK1/2) inhibitor, to treat RAIR-DTC. Four out of nine patients with BRAF mutations exhibited RAI uptake, though only one exceeded the threshold for RAI treatment. In contrast, all five patients with NRAS mutations achieved RAI uptake levels above the therapeutic threshold. This study marked a pivotal advancement in redifferentiation strategies using MAPK inhibitors. To further evaluate selumetinib’s efficacy, Brown et al. conducted a single-arm, multicenter phase II trial (SEL-I-METRY), enrolling 28 RAIR patients who received selumetinib at 75 mg twice daily for four weeks [51]. Nine patients demonstrated significant RAI uptake and were treated with RAI, with two remaining progression-free at the final follow-up.

Given the relatively poor response of selumetinib in BRAF-mutant thyroid cancer, other MAPK inhibitors have been explored for redifferentiation strategies [15, 16, 52–58]. Rothenberg et al. investigated the redifferentiation potential of dabrafenib, a selective BRAF inhibitor, in patients with BRAF-mutant PTC [16]. Six of the ten patients showed new RAI uptake on whole-body scans, with two achieving partial response (PR) and four showing stable disease (SD) after 3 months. Similarly, Dunn et al. found that vemurafenib increased RAI uptake in RAIR patients with BRAF-mutation [15]. Additionally, the study noted that higher baseline Tg levels in responders may indicate that tumor differentiation status could predict vemurafenib efficacy. In conclusion, redifferentiation therapies offer promising potential, providing the possibility of prolonged remission or even a cure for the disease.

With the advancement of immunotherapy, immune checkpoint inhibitors (ICIs), such as PD-1/PD-L1 inhibitors, are being explored as potential strategies in RAIR treatment [59–64]. In a phase Ib trial, pembrolizumab demonstrated limited antitumor activity against advanced DTC expressing PD-L1, with overall efficacy being suboptimal [59]. Of the 22 patients treated, 2 achieved PR and 13 SD, with a median PFS of 7 months. Building on these findings, researchers have investigated combination treatments with ICIs. French et al. reported results from a study evaluating lenvatinib plus pembrolizumab in RAIR patients who were either treatment-naive to multi-kinase inhibitors (cohort 1) or had progressed on lenvatinib (cohort 2) [64]. In cohort 1, the ORR was 65.5%, with a median PFS of 26.8 months, likely influenced by lenvatinib's efficacy. In cohort 2, the ORR was 16%, but disease control reached 96%, with 36% of patients showing no disease progression at 15 months. Importantly, no increase in toxicity was observed with the combination therapy, suggesting that combining TKIs with immunosuppressants may be a viable salvage therapy. This promising area of research warrants further attention and investment.

### Green cluster: debates surrounding the RAIR-DTC management

In the yellow cluster, prominent keywords, such as "management," "association guidelines," and "prognosis", emphasize the importance of developing guidelines grounded in the latest scientific evidence. As RAIR-DTC treatment continues to advance, authorities are regularly revising and updating guidelines to offer scientifically sound and comprehensive recommendations for medical practice worldwide [5, 10, 65–69].

The American Thyroid Association (ATA) guidelines hold significant influence globally. Studies have shown that trends in thyroid cancer incidence and RAI treatment closely align with updates to the ATA guidelines, demonstrating the swift adaptation of medical practice to new research findings [70–73]. Dr. Haugen, a key contributor to the ATA guidelines, has emphasized the importance of new or revised recommendations [30]. The guidelines have received widespread recognition for their clarity and accessibility, making them easier to understand and apply to a broad audience, including clinicians, researchers, and patients.

The promotion and application of the ATA guidelines have sparked debate and discussion among various academic groups. Both the European Association of Nuclear Medicine (EANM) and the Society for Nuclear Medicine and Molecular Imaging (SNMMI) have raised concerns about certain aspects of the ATA guidelines [74]. In 2019, the European Thyroid Association (ETA) published its own guidelines for the treatment and follow-up of advanced RAIR-DTC, redefining RAIR-DTC and addressing critical topics, such as treatment timing (particularly for multi-kinase inhibitors therapy), dosage, and follow-up strategies [67]. To resolve these differences and build consensus, the ATA, ETA, EANM, and SNMMI issued a joint statement in April of that year, known as the Martinique Principles [74]. This statement outlines five common "clinical scenarios" for RAIR-DTC, emphasizing that the definition is not absolute and encouraging clinicians to personalize treatment based on individual patient needs. This initiative represents a significant step forward in international collaboration and consensus-building in RAIR-DTC management. As guidelines continue to be updated, the management of RAIR-DTC is expected to become increasingly scientific and precise.

In 2023, Liu et al. investigated the predictive value of blood biomarkers for RAIR-DTC in a case–control study involving 36 patients [75]. The findings revealed that the ratio of low-density lipoprotein cholesterol to total cholesterol (LDL-Ch/Tch) and white blood cell (WBC) levels at the time of surgery could predict RAIR, with a combined prediction model achieving an AUC of 0.861. A year later, Schubert et al. conducted a risk factor analysis on 159 RAIR patients and 759 controls [76]. Their study identified seven independent risk factors for the development of RAIR in DTC patients: age at diagnosis ≥ 55, vascular invasion, synchronous cervical, pulmonary, and bone metastases at initial work-up, and cervical and pulmonary recurrence during follow-up. Early identification of RAIR is crucial for optimizing therapeutic strategies and improving patient outcomes and quality of life [77]. Therefore, continued research in this area holds great promise for developing more precise diagnostic tools and enabling personalized treatment strategies for RAIR patients.

This study has several limitations. First, it only includes articles from the WoSCC database. While WoSCC covers most high-quality research, this may limit the inclusion of relevant studies from other databases. Second, recently published high-quality articles may have low citation counts due to their shorter publication time, which could affect their visibility and influence in the analysis.

## Conclusions

Since the twenty-first century, the number of publications in the field of RAIR-DTC treatment has been on the rise overall. The United States has emerged as the foremost contributor to scientific research in this area. Current research hotspots include immunotherapy and prognosis, with a particular focus on combination therapy involving ICIs and other medications. Equally important, the redifferentiation strategy creates an opportunity for RAIR-DTC patients to be reintroduced into RAI therapy. Therefore, there is an urgent need for robust phase 2/3 trials to validate the efficacy and safety of these treatments. Furthermore, novel biomarkers should be actively explored to identify RAIR-DTC patients as early and accurately as possible, thereby gaining more time for the implementation of effective treatment regimens. Our study provides insights into historical and current trends in RAIR-DTC treatment research, which may inform future drug development, disease diagnosis, and the exploration of combination therapies.
